# Rapid Adsorption of Heavy Metals by Fe_3_O_4_/Talc Nanocomposite and Optimization Study Using Response Surface Methodology

**DOI:** 10.3390/ijms150712913

**Published:** 2014-07-21

**Authors:** Katayoon Kalantari, Mansor B. Ahmad, Hamid Reza Fard Masoumi, Kamyar Shameli, Mahiran Basri, Roshanak Khandanlou

**Affiliations:** Department of Chemistry, Faculty of Science, Universiti Putra Malaysia, Serdang 43400, Malaysia; E-Mails: Fardmasoumi@upm.edu.my (H.R.F.M.); kamyarshameli@gmail.com (K.S.); mahiran@upm.edu.my (M.B.); roshanak_bch@yahoo.com (R.K.)

**Keywords:** heavy metals, Fe_3_O_4_/talc nanocomposites, adsorption, response surface methodology (RSM), adsorption kinetics

## Abstract

Fe_3_O_4_/talc nanocomposite was used for removal of Cu(II), Ni(II), and Pb(II) ions from aqueous solutions. Experiments were designed by response surface methodology (RSM) and a quadratic model was used to predict the variables. The adsorption parameters such as adsorbent dosage, removal time, and initial ion concentration were used as the independent variables and their effects on heavy metal ion removal were investigated. Analysis of variance was incorporated to judge the adequacy of the models. Optimal conditions with initial heavy metal ion concentration of 100, 92 and 270 mg/L, 120 s of removal time and 0.12 g of adsorbent amount resulted in 72.15%, 50.23%, and 91.35% removal efficiency for Cu(II), Ni(II), and Pb(II), respectively. The predictions of the model were in good agreement with experimental results and the Fe_3_O_4_/talc nanocomposite was successfully used to remove heavy metals from aqueous solutions.

## 1. Introduction

The release of heavy metals in aqueous systems is of severe concern due to their hazardous effects on human and the environment. Heavy metal pollution exists in the aqueous waste stream from several industries such as metal plating, battery manufacture, pharmaceutical, mining, tanneries, and painting, as well as farming sources where fertilizers and fungicidal spray are intensively applied [1]. Several conventional techniques have been reported to remove metal ions from aqueous solutions, such as oxidation, reduction, precipitation, membrane filtration, ion exchange, and adsorption. Among these methods, adsorption is the most favorable process, economically and technically, for removing heavy metals from aqueous solutions [2,3]. Recently, Fe_3_O_4_ nanoparticles were shown to be highly efficient materials for heavy metal ion removal by adsorption; metal ion adsorption by magnetite was demonstrated through a combination of electrostatic attraction and ligand exchange [4,5,6]. Fe_3_O_4_ nanoparticles can be rapidly and easily separated from aqueous solutions using an external magnetic field due to their magnetic property and have some advantages of sensitivity and high efficiency. Therefore, adding Fe_3_O_4_ nanoparticles to the adsorbent is an excellent way to resolve separation problems [7], but there are some challenges that Fe_3_O_4_ nanoparticles present. The first one is that Fe_3_O_4_ nanoparticles oxidize and dissolve easily. Also, the recycling process is difficult due to the small size of nanoparticles. Finally, the nanoparticles tend to co-aggregate and thus the effective surface area decreases, reducing their reaction activity. In order to protect Fe_3_O_4_ nanoparticles, metal, polymer and a silica shell were used [8]. Several techniques have been developed to minimize the co-aggregation of the Fe_3_O_4_ nanoparticles and improve their manipulation, for example using polymers and clays [9]. Moreover, clay soils are widely used as adsorbents that isolate hazardous and other waste materials from surrounding environments.

There are few studies on the adsorption characteristics of heavy metal ions in talc. Talc is known by the chemical formula Mg_3_Si_4_O_10_(OH)_2_. It consists of a magnesium hydroxide layer (MgO∙H_2_O) sandwiched between two silicate layers (SiO_2_), forming a three-layer structure [10]. Adjacent layers are connected by weak van der Waals forces, providing talc a platy structure. The low energy silicate layers of talc planes, [001] crystal domains, have hydrophobic properties, while the edges displaying the hydroxyl groups (–SiOH) and (–MgOH) are more hydrophilic [11,12]. Talc is commonly used as a filler, coating and dusting agent in paints, lubricants, plastics, cosmetics, pharmaceuticals, and ceramics manufacture [13]. In this work, Fe_3_O_4_/talc nanocomposite was used for removal of Cu(II), Ni(II) and Pb(II) ions from aqueous solutions and response surface methodology (RSM) was applied for optimization study and screening variables effects on ion removal.

In traditional methods, one variable at a time is employed for monitoring the effect of functional variables. In this optimization method, the analyzed parameter is changed; others are kept at a fixed level. This technique cannot evaluate interactive effects between the variables and uses a large number of experiments, which is time consuming and costly [14]. Multivariate statistical methods have been preferred to identify the perfect combination of factors and interactions among elements, which are not possible to recognize using the one variety method [15] . Additionally, these techniques are very beneficial tools to save the time and reduce the cost of research. The actual design consists of estimation of the coefficients in a mathematical model, predicting the response, and checking the adequacy of the model. Essentially the widely used designs to find out response surfaces are factorial designs and the more complex response surface methodologies [16,17,18].

In the present work, the ability of a hybrid material consisting of the talc sheets as support of magnetite particles, for removal of Cu(II), Ni(II), and Pb(II) from aqueous solutions was studied. The metal adsorption capacity of the talc can be manipulated by adding Fe_3_O_4_ nanoparticles, which increase the amount of heavy metal uptake. The adsorption experiments were performed and the influence of heavy metals ion concentration, removal time and adsorbent amount were analyzed by the response surface methodology. To the best of our knowledge, there are not examples in the literature dealing with the removal of Cu(II), Ni(II) and Pb(II) by Fe_3_O_4_/talc nanocomposite as an adsorbent. Furthermore, the synthesized of magnetite/talc nanocomposite has not been reported except in our previous study [19]. The objective of this study was therefore to examine the ability of Fe_3_O_4_/talc nanocomposite to remove heavy metal ions from aqueous solution according to response surface methodology design.

## 2. Results and Discussion

### 2.1. Brunauer–Emmett–Teller (BET) Surface Area

Specific surface area of the talc powder and Fe_3_O_4_/talc nanocomposite were determined by the Brunauer–Emmett–Teller (BET) method (acquisition and reduction). The BET isotherm is the basis for determining the extent of nitrogen adsorption on a given surface. A Quantachrom AS1Win 2008 (Quantachrome Instruments, Boynton Beach, FL, USA) was used in this work for measuring the surface area of samples. The systematic sorption and desorption of nitrogen provided the fundamental information on the surface characteristics. The surface areas of talc powder and Fe_3_O_4_/talc nanocomposite were found to be 6.675 and 37.079 m^2^/g, respectively.

### 2.2. Modeling of Adsorption Process

#### 2.2.1. Central Composite Rotatable Design (CCRD)

The Central Composite Rotatable Design (CCRD) was used to determine the influence of experimental variables and the interactions on the heavy metal ions removal. The CCRD had eight factorial points, six axial points, and six center points, resulting in a total of 20 runs used to determine of optimum points. Table 1 provides a summary of selected experimental parameters and their values. All experiments were carried out in actual pH to prevent precipitation. The quantity of metal ion removal (*Y*) was taken as the response of the design experiments.

The relationship between the removal of heavy metal ions and the factors was obtained with coded variables as the following for Cu(II), Ni(II), and Pb(II), respectively:

Cu(II) removal (%) = 48.03 − 14.99*A* + 0.11*B* + 4.73*C* − 1.25*AB* + 0.37*AC* + 1.00*BC* + 2.97*A*^2^ − 0.12*B*^2^ + 1.38*C*^2^ + 0.63*ABC* (1)

Ni(II) removal (%) = 28.77 − 7.36*A* + 1.43*B* + 1.88*C* − 2.04*AB* + 0.12*AC* + 3.84*BC* + 3.37*A*^2^ + 0.69*B*^2^ − 0.62*C*^2^ − 1.64*ABC* (2)

Pb(II) removal (%) = 89.25 − 6.44*A* + 2.40*B* − 0.44*C* + 2.02*AB* + 5.37*AC* + 3.66*BC* − 9.62*A*^2^ + 1.40*B*^2^ + 0.33*C*^2^ + 0.53*ABC* (3)

where *A* is the initial concentration of heavy metal ion coefficient, *B* the time coefficients, and *C* the adsorbent dosage coefficients, expressed as experimental variables. Good correlation between the experimental results and the predicted values (Equations (1)–(3)) illustrates that the designs are properly fitted (*R*^2^ > 0.97 for all three models). For the validation objective, experiments were conducted for 6 new trials, consisting of combinations of experimental factors, which do not examine the training data set. The actual and predicted values are presented in Table 2. The comparison between experimental observed and predicted data shows excellent agreement for all heavy metal ion removals.

**Table 1:** Predicted (Pre.) and experimental (Exp.) design matrix obtained by CCRD.

Run No.	Initial Ion Concentration (mg/L)	Removal Time (s)	Adsorbent Dosage (g)	Removal of Cu(II) (%)		Removal of Ni(II) (%)		Removal of Pb(II) (%)	
				Exp.	Pre.	Exp.	Pre.	Exp.	Pre.
1	100	40	0.08	62.50	61.92	38.89	39.82	83.38	82.66
2	200	13	0.10	47.50	47.51	30.43	28.31	89.49	89.17
3	100	120	0.08	64.00	63.89	36.94	35.80	90.20	90.60
4	200	80	0.10	49.00	48.03	28.54	28.77	88.55	89.25
5	100	120	0.12	73.00	73.35	51.31	50.30	83.44	85.23
6	100	40	0.12	70.00	69.88	31.31	32.37	76.81	78.20
7	32	80	0.10	81.00	81.65	50.82	50.67	74.51	72.91
8	200	80	0.07	44.00	43.99	23.38	23.87	89.23	90.09
9	200	80	0.10	43.00	48.03	27.31	28.77	87.02	89.25
10	200	80	0.10	51.00	48.03	30.68	28.77	88.67	89.25
11	300	120	0.12	41.50	42.87	29.86	28.44	87.00	88.13
12	300	40	0.12	41.00	41.90	24.62	25.27	70.19	70.91
13	200	80	0.13	61.00	59.90	30.00	30.20	91.05	88.60
14	300	120	0.08	28.50	29.41	21.59	20.04	70.17	69.90
15	368	80	0.10	33.00	31.23	25.08	25.91	69.69	68.46
16	200	80	0.10	46.00	48.03	28.70	28.77	92.11	89.25
17	300	40	0.08	34.50	34.94	25.14	25.66	70.10	69.43
18	200	147	0.10	49.00	47.88	30.32	33.13	98.53	97.26
19	200	80	0.10	52.00	48.03	28.84	28.77	88.36	89.25
20	200	80	0.10	47.00	48.03	28.70	28.77	90.53	89.25

**Table 2:** Validation set.

Initial Ion Concentration (mg/L)	Removal Time (s)	Adsorbent Dosage (g)	Cu(II) Removal %		Ni(II) Removal %		Pb(II) Removal %	
			Exp.	Pre.	Exp.	Pre.	Exp.	Pre.
150	80	0.1	55.64	56.27	33.35	32.29	93.64	92.09
250	80	0.1	42.19	41.27	24.75	25.93	80.67	78.58
200	60	0.1	46.78	47.94	29.88	28.22	82.52	81.41
200	105	0.12	55.11	54.79	32.65	33.60	90.14	88.71
200	80	0.06	43.68	44.10	23.06	22.54	83.78	82.99
200	80	0.11	51.43	50.74	30.15	29.56	84.29	85.26

**Figure 1:**
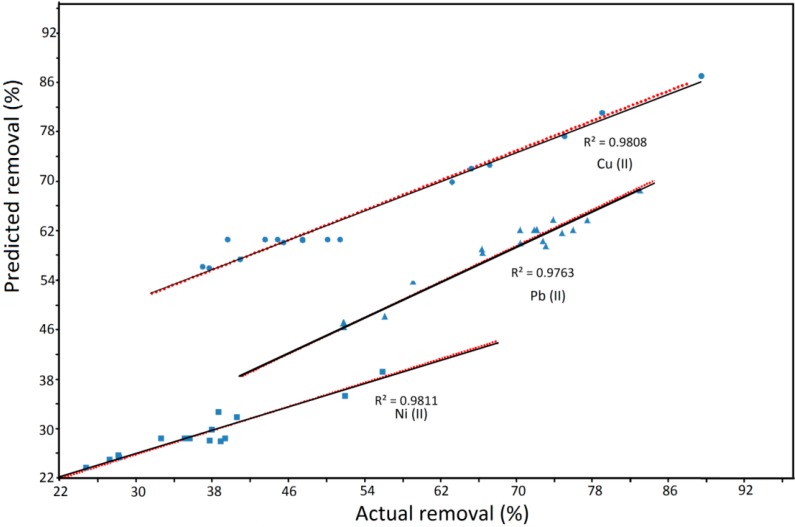
Actual results *versus* predicted results. Solid lines: experimental results; dashed lines: Predicted results.

Figure 1 represents the assessment of predicted and actual results. It is clearly recognized that the predicted values were fitted well with the actual values. The coefficients of determination for Cu(II), Ni(II) and Pb(II) removal were obtained 0.9808, 0.9811 and 0.9763, respectively. As shown in Figure 1, the responses predicted from RSM were compared to the actual values, for the verification of the predicted data.

The analysis of variance (ANOVA) for Cu(II), Ni(II) and Pb(II) removal was used to estimate the response of initial concentration of heavy metal ion (mg/L), removal time (s) and dosage of adsorbent (g) as shown in Table 3.

The *F* values of the design for Cu(II), Ni(II) and Pb(II) removal were 48.22, 36.73 and 40.10, respectively, and demonstrate that the models were statistically significant and there was only a 0.01% chance in Cu(II) and Ni(II) removal and 0.02% in Pb(II) removal that the model *F* values could incur due to noise. The lack of fit *F* value of Cu(II) removal is 0.23 and implied that it was not significant relative to the pure error. A non-significant lack of fit was considered good and was desired for the model to fit. Also, for the Ni(II) and Pb(II) removal, the lack of fit *F* values of 4.86 and 0.17 implied that they were not significant relative to the pure error for these metal ions removals and that the quadratic models were valid for the present study.

As demonstrated in Table 3, the coefficients of determination (*R*^2^) are found to be 0.9817, 0.9761 and 0.9804 for Cu(II), Ni(II) and Pb(II) removal, respectively. Obviously, the value of *R*^2^ should be between zero and 1 (0 ≤ *R*^2^ ≤ 1) and the larger amounts are better. The quantity “Std.Dev” represents the square root of the variance. The term PRESS means that the predicted residual sum of squares is used as a criterion for the model’s efficiency to predict the responses of a new experiment. The smaller levels of PRESS are more suitable.

**Table 3:** Analysis of variance of the fitted quadratic equation and model summary statistics for removal% of Cu(II), Ni(II) and Pb(II); *A*, Initial ion concentration (mg/L); *B*, Removal time (s); *C*, Adsorbent dosage (g); PRESS, Predicted residual sum of squares.

Source	Removal of Cu(II) (%)			Removal of Ni(II) (%)			Removal of Pb(II) (%)		
	Mean Square	*F*-Value	*p*-Value	Mean Square	*F*-Value	*p*-Value	Mean Square	*F*-Value	*p*-Value
Model	354.79	48.22	<0.0001	116.90	36.73	<0.0001	251.59	40.10	<0.0001
*A*	3068.99	417.14	<0.0001	740.02	232.52	<0.0001	1837.38	292.88	<0.0001
*B*	0.17	0.023	0.8826	28.02	8.81	0.0158	422.79	67.39	<0.0001
*C*	305.48	41.52	0.0001	48.36	15.20	0.0036	14.78	2.36	0.5891
*AB*	12.50	1.70	0.2248	33.45	10.51	0.0101	112.59	17.95	0.1634
*AC*	1.13	0.15	0.7049	0.11	0.036	0.8536	65.65	10.46	0.0029
*BC*	8.00	1.09	0.3243	118.13	37.12	0.0002	157.80	25.15	0.0120
*A*^2^	127.45	17.32	0.0024	163.22	51.29	<0.0001	173.29	27.62	0.0010
*B*^2^	0.21	0.028	0.8706	6.85	2.15	0.1765	543.84	86.69	0.0008
*C*^2^	27.56	3.75	0.0849	5.46	1.72	0.2227	27.41	4.37	<0.0001
*ABC*	3.13	0.42	0.5309	21.64	6.80	0.0284	265.58	42.33	0.0700
Residual	7.36	–	–	3.18	–	–	6.27	–	–
Lack of fit	2.55	0.23	0.9115	5.70	4.86	0.0565	1.57	0.17	0.9108
Pure error	11.20	–	–	1.17	–	–	9.10	–	–
Standard deviation	2.71			1.78			2.50		
PRESS	218.99			318.77			164.81		
*R*^2^	0.9817			0.9761			0.9804		
Adjusted *R*^2^	0.9613			0.9495			0.9560		
Predicted *R*^2^	0.9394			0.7338			0.9359		
Adequate precision	25.972			23.153			23.846		

#### 2.2.2. 3D Response Surface Plots

**Figure 2:**
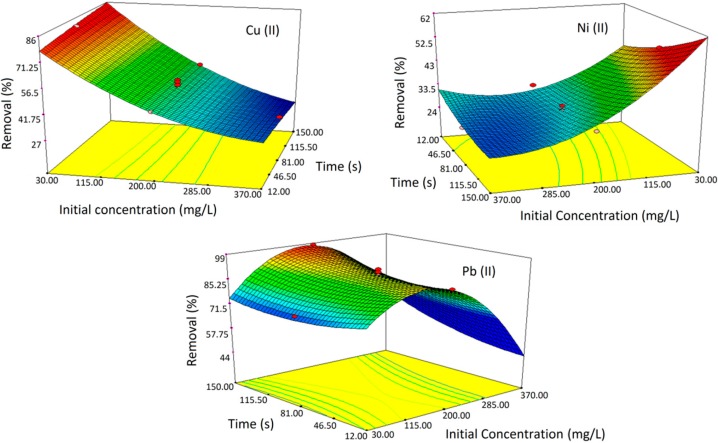
Response surface 3D plots indicating the effect of interaction between heavy metal ion concentration and removal time for Cu(II), Ni(II) and Pb(II).

**Figure 3:**
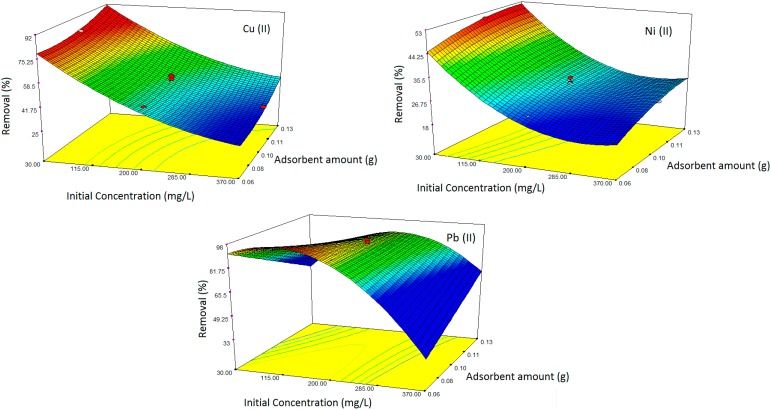
Response surface 3D plots indicating the effect of interaction between heavy metal ion concentration and adsorbent amount for Cu(II), Ni(II) and Pb(II).

Figure 2 and Figure 3 demonstrate the response surface 3D plots for the effect of interaction between time, initial ion concentration and amount of adsorbent for Cu(II), Ni(II) and Pb(II) ion removal, respectively.

As mentioned in Figure 2, the effect of removal time was not particularly critical in improving the sorption capacity of nanocomposite, while the concentration of the heavy metal ions confirmed a stronger effect on the adsorption efficiency. It is realized that through moving towards the initial ion concentration axis at a constant time, the removal values decreased dramatically. The trend of Pb(II) in Figure 2 demonstrates that after saturation level, the removal efficiency decreased since the sites are covered with metal ions. The design predicted for Cu(II) and Ni(II) removal similarly shows that the removal efficiency is decreased with increases in concentration past saturation level [20]. As shown in Figure 3, the adsorbent dosage plays an important role in the removal efficiency and clearly demonstrates that with increasing the adsorbent dosage, removal efficiency improves. This could be explained due to the higher amounts of adsorbent because of an increase in the adsorbing surface and that the magnetically active surfaces prepare some surfaces for adsorbing metal ions. The fast adsorption by Fe_3_O_4_ nanoparticles is probably attributed to the exterior surface adsorption. All the adsorption sites of Fe_3_O_4_ nanoparticles can be found on the exterior of the adsorbent; it is possible for the adsorbate (ion) to get into these active sites, thus causing a rapid approach to equilibrium [5]. As a result, sets of solution were produced with the software for the optimum conditions of the adsorption of heavy metal ions that are described in Table 4. These experiments demonstrate that both results were in good agreement.

**Table 4:** Optimized conditions for Cu(II), Ni(II) and Pb(II) removal. Std.Dev, the square root of the variance.

Metal	Initial Ion Concentration (mg/L)	Removal Time (s)	Adsorbent Dosage (g)	Removal (%)			
				Actual	Predicted	Error	Std.Dev
Cu(II)	100	120	0.12	72.15	73.35	1.2	0.84
Ni(II)	92	120	0.12	50.23	51.64	1.41	0.99
Pb(II)	270	120	0.12	91.35	92.15	0.8	0.56

The difference within the adsorption possibly results from differences in the radius of Cu(II), Ni(II) and Pb(II) ions. The heavy metal ion removal efficiency followed the increasing order: Pb(II) > Cu(II) > Ni(II). Since the radius of Ni(II) (0.69 Å) is noticeably smaller than that of Cu(II) (0.73 Å) and Pb(II) (1.32 Å), nickel ions are easier to hydrate than Pb(II), therefore forming a larger water layer on the surface. As a result, Ni(II) and Cu(II) are more mobile in bulk solution and would have a lesser tendency to adsorb on the nanoadsorbent [21].

#### 2.2.3. Sorption Isotherms

Two different isotherms that are widely used for the adsorption processes are Langmuir and Freundlich isotherms. The precision of these isotherms to simulate experimental data is greatly affected by the particular sorbate-sorbent system [22]. The Langmuir isotherm is generally more appropriate to monolayer adsorption and all metal binding sites are energetically the same and there are neither interactions between adsorbed molecules nor the transmigration of sorbate in the plane of the surface area [23]. On the other hand, the Freundlich isotherm may be used for non-ideal sorption which involves heterogeneous sorption [24].

The Langmuir equation is expressed by the following expression:

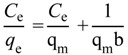
(4)
where *C*_e_ is the equilibrium concentration of the solute (mg/L), q_m_ is the maximum adsorption capacity (mg/g), the amount adsorbed at equilibrium is *q_e_* (mg/g) and b (L·mg^−^^1^) is the Langmuir constant, respectively [25]. The values of q_m_ and b are determined from the slope and intercept of the linear plots of *C*_e_/*q*_e_ *versus* *C*_e_, respectively.

**Figure 4:**
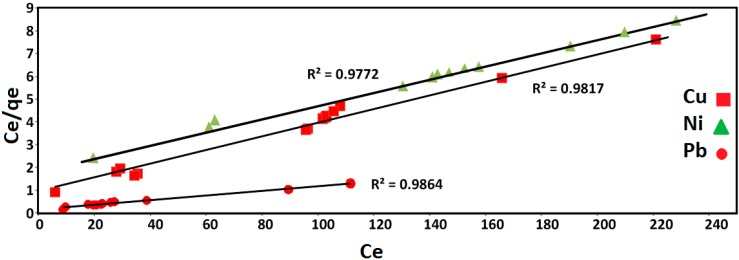
Langmuir isotherm fitting by the Fe_3_O_4_/talc nanocomposite for Cu(II), Ni(II) and Pb(II) removal process.

Figure 4 shows the Langmuir isotherm fitting of Cu(II), Ni(II) and Pb(II) adsorption. The result shows satisfactory fitting to the experimental data for all metal ions with *R*^2^ as 0.9817, 0.9772 and 0.9864 for Cu(II), Ni(II) and Pb(II), respectively.

The Freundlich equation is expressed as:

*q_e_* = *K_F_ C_e_^1/n^* (5)

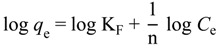
(6)
where K_F_ and 1/n relate the adsorbent capacity and sorption intensity of the adsorbent, respectively. The higher *R*^2^ values obtained as shown in Table 5 indicate that the experimental data obeyed the Langmuir isotherm more than the Freundlich isotherm.

**Table 5:** Langmuir and Freundlich constant values for the heavy metal ion removal.

Isotherm	Langmuir			Freundlich		
	*R*^2^	b	q_m_	*R*^2^	n	K_F_
Cu(II)	0.9817	0.017	21.05	0.9620	2.435	1.725
Ni(II)	0.9772	1.061	33.33	0.9601	1.752	1.150
Pb(II)	0.9864	5.042	74.62	0.9032	3.109	3.287

#### 2.2.4. Kinetics Studies

**Figure 5:**
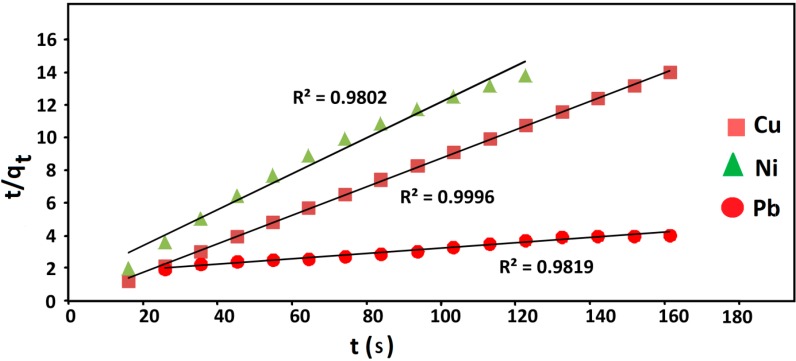
Kinetics models for the adsorption of Cu(II), Ni(II) and Pb(II) on Fe_3_O_4_/talc nanocomposite.

The kinetic study of adsorption processes provides useful data concerning the efficiency of the adsorption and the feasibility for scale-up operations. The kinetic data of adsorption can be examined using various types of mathematic designs, of which one most widely used is Lagergren’s rate equation [26]. The kinetics of the adsorption process was analyzed using the pseudo-second order rate equation given by:

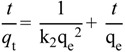
(7)
where k_2_ is the pseudo-second order rate constant (g/(mg·min)). The q_e_ and k_2_ can be obtained by linear plot of *t*/*q*_t_ versus *t* [27]. Kinetics studies for the adsorption of Cu(II), Ni(II) and Pb(II) on Fe_3_O_4_/talc nanocomposite were performed using pseudo-first order and pseudo-second order kinetic models. Pseudo-second order kinetic plot of *t*/*q*_t_ *versus* *t* provided the perfect straight line for the adsorption of all metal ions onto adsorbent indicating that adsorption reaction can be estimated with pseudo-second order kinetic model which is demonstrated in Figure 5.

The values of design parameters k_1_, k_2_, q_e_ and correlation coefficient (*R^2^*) are obtained from the plots and presented in Table 6. As demonstrated in this table, the correlation coefficients of the second order rate equation, in all the metal ions adsorptions, are near the 0.99 and significantly higher than that for the first order rate equation. Also, the q_e_ values calculated from the second order kinetics model agree well with the experimental values. This shows that the adsorption of copper, nickel and lead ions can be displayed by the pseudo-second order model.

**Table 6:** Parameter of the kinetics models for the adsorption of Cu(II), Ni(II) and Pb(II) onto Fe_3_O_4_/talc nanocomposite.

Heavy Metal Ions	First Order		Second Order		
	*R*^2^	k_1_	*R*^2^	k_2_	h
Cu(II)	0.9702	0.01	0.9996	1.42 × 10^−2^	0.1607
Ni(II)	0.8695	0.008	0.9802	2.43 × 10^−3^	0.1872
Pb(II)	0.8814	0.0489	0.9819	2.22 × 10^−4^	3.1736

The rate constant of pseudo-second order adsorption (k_2_) obtained for Pb(II) removal was found to be lower than that computed for Ni(II) and Cu(II). This indicated the uptake of Pb(II) onto nano adsorbent from aqueous solution was more rapid and favorable. It can be observed that the initial adsorption rate, *h* (mg/(g·min)), is higher for Pb(II) than Ni(II) and Cu(II). This is an indication that initial adsorption of Pb(II) by nano adsorbent was faster in comparison with other metal ions, and that Pb(II) may be quantitatively removed earlier.

#### 2.2.5. Adsorption Studies

The particular properties of the nanoadsorbent (porous and surface structure, *etc.*) are essential in this process. The individual influences of adsorbent amount, removal time and initial ion concentration on heavy metals removal were analyzed. The uptake of heavy metal ions improved with increase in adsorbent dosage because higher numbers of vacant sites on the surface area were available for adsorption [28]. Additionally, the results illustrate that with increasing heavy metal ion concentration, the removal efficiency decreased; this can be explained due to the fact that the remaining vacant sites are difficult be filled with the heavy metal ions because of repulsive forces between the adsorbed solute molecules on the surface and solute in bulk phase. When talc sheet are broken, two different surfaces are created: one resulting from the easy cleavage of the layers, known as “faces” composed of completely compensated oxygen atoms, present at a very low electrical charge and nonpolar in water and hydrophobic; and the other arising from the break of the ionic bonds within the layers, named “edges” made up of hydroxyl ions, siloxane groups (–Si–O–Si–), oxygen, and magnesium ions that simply undergo hydrolysis, providing a comparatively high electrical charge, and are polar in water, but functional hydroxyl groups such as –SiOH, and –MgOH belonging to edge surfaces cause a hydrophilic effect [29].

The negative hydrophilic surface sites of talc particles (≡SiO^−^) are investigated by adsorption of cationic molecules used as molecular probes. The electrostatic attraction between opposite charges of metals and negative surface sites on talc leads to adsorption and suggests a strong affinity. Metal ions can be bonded to edge surfaces of talc sheets using hydroxyl groups or with isomorphous centers of substitution centers of Si^4+^ ions for Al^3+^ ions on basal planes [30].

According to BET results, Fe_3_O_4_ nanoparticles provide higher surface area in nanocomposites compared to pure talc powder, and increase the ability of talc in heavy metal ion adsorption [31]. Furthermore, the metal ions all rapidly adsorbed at less than 2 min, so fast binding of metal ions to Fe(III) species at the external surface and quick access by metals can be mentioned as a subsidiary mechanism [32].

## 3. Experimental Section

### 3.1. Materials and Methods

All reagents in this work were of analytical grade and used as received without further purification. Ferric chloride hexahydrate (FeCl_3_·6H_2_O) and ferrous chloride tetrahydrate (FeCl_2_·4H_2_O) of 96% were used as the iron precursor and also, talc powder (<10 μg, 3MgO·4SiO_2_·H_2_O) were obtained from Sigma-Aldrich (St Louis, MO, USA). NaOH of 99% was obtained from Merck KGaA (Darmstadt, Germany). Copper chloride, lead nitrate (II), and nickel chloride were supplied by Hamburg Chemical (Hamburg, Germany). All aqueous solutions were prepared with deionized water.

### 3.2. Fe_3_O_4_/Talc Nanocomposites Preparation

The chemical co-precipitation technique has been used in preparation of nano particles. For the synthesis of Fe_3_O_4_/talc nanocomposites, 2.0 g of talc was suspended in 120 mL deionized water, and then a solution of Fe^3+^ and Fe^2+^ with (2:1) molar ratio was added into the mixture. The ion solution suspended with talc composites were stirred for 24 h for impregnation by the external surface of talc layers to prepare talc/Fe^3+^–Fe^2+^ composites. Then 25 mL of freshly prepared NaOH (2.0 M) was added to talc/Fe^3+^–Fe^2+^ composites suspension under continuous stirring. The suspensions were finally centrifuged, washed twice with ethanol and distilled water, and kept in a vacuum stove at 100 °C. All the experiments were conducted at an ambient temperature and under a non-oxidizing oxygen free environment through the flow of nitrogen gas [33].

### 3.3. Adsorbate

Three stock solutions (1000 mg/L) of Cu(II), Ni(II) and Pb(II) ions were prepared by dissolving appropriate amounts of copper chloride, nickel chloride, and lead nitrate in deionized water and then transferred to 1-liter volumetric flasks and diluted with deionized water. The stock solutions were then diluted with deionized water to obtain the desired concentration range of Cu(II), Ni(II) and Pb(II) standard solutions.

### 3.4. Experimental Procedures

Different amount of nano-adsorbent according to RSM design was mixed with 25 mL solution of single metallic ions of Cu(II), Ni(II) and Pb(II) using a shaker at the temperature of 25 °C. After that, the suspension was magnetically separated from the aqueous solution and the residual concentration of metal ions in the solution was analyzed. The heavy metal ion concentration in the filtrate was determined using AAS (Atomic Absorption Spectrophotometry, S Series; Thermo Scientific, Waltham, MA, USA).

The response removal efficiency of heavy metal ions was calculated as Equation (8):

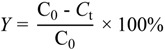
(8)
where *Y* is the percentage of adsorption, C_0_ is the initial concentration of heavy metal ions (mg/L) and *C*_t_ is the concentration of heavy metal ions at time *t*.

All experiments were carried out in triplicate and the mean values are reported. The maximum deviation was found to be ±2%.

## 4. Conclusions

In this work, a new nanoadsorbent was successfully used for heavy metal removal. Such synthesized adsorbent has not only a unique structure with a large surface area, but also a superparamagnetic character. These features make it an effective and convenient adsorbent for heavy metals removal. Response surface methodology and central composite rotary design were appreciable in determining the optimal conditions for adsorption. In addition, the amount of sorption of metal ions on nanoadsorbent increased with increasing adsorbent dosage. The adsorption kinetics abides by pseudo second order kinetic equation, and the Langmuir isotherm fitted well with the adsorption process. The prepared adsorbent performed in neutral actual pH condition to prevent precipitation. In addition, this adsorbent could be adsorbed by an external magnetic field after heavy metal ion adsorbing. A rapid sorption of Cu(II), Ni(II) and Pb(II) was found on the Fe_3_O_4_/talc nanocomposite during less than 2 min. Moreover, In aqueous solutions, the high concentrations of heavy metal ions (100, 92 and 270 mg/L) for Cu(II), Ni(II) and Pb(II), respectively were adsorbed in the very low level amount of adsorbent (around 0.12 g).

The actual results were in good agreement with the predicted data by models. Experimental results show that the use of Fe_3_O_4_ nanoparticles for the heavy metal ion removal is technically achievable, environmentally friendly, and economically attractive for the treatment of water. Compared to conventional separation, the advantages of adsorption followed by magnetic separation are attributed to its rapidness, effectiveness, and simplicity.
